# A Comparison of the Application of Two Different Scoring Systems in a Patient With Upper Limb Necrotizing Fasciitis

**DOI:** 10.7759/cureus.61682

**Published:** 2024-06-04

**Authors:** Saskah Thompson, Keonne Cooper, Thalia Thomas, Kimberly Alexander, Shivaughn Hem-Lee-Forsyth

**Affiliations:** 1 Emergency Medicine, Eastern Regional Health Authority, Sangre Grande, TTO; 2 Orthopedic Surgery, Eastern Regional Health Authority, Sangre Grande, TTO; 3 General Surgery, Eastern Regional Health Authority, Sangre Grande, TTO; 4 Public Health, St. George's University, St. George's, GRD

**Keywords:** siari, lrinec, surgical debridement, predictor tools, scoring system, necrotizing fasciitis

## Abstract

Necrotizing fasciitis (NF) is a life-threatening soft-tissue infection that requires early recognition and surgical debridement to ensure the best outcome for patients. The Laboratory Risk Indicator for Necrotizing Fasciitis (LRINEC) score and the SIARI (Site other than lower limb, Immunosuppression, Age <60 years, Renal Impairment and Inflammatory markers) score are clinical predictor tools that can aid in the timely diagnosis of NF. This case report discusses a male patient who presented with a rash on his arm that was initially thought to be cellulitis. It examines how the application of scoring systems can be beneficial for earlier identification or when the diagnosis is uncertain.

## Introduction

Necrotizing fasciitis (NF) is a rare infection affecting the subcutaneous tissue and fascia of the body. It progresses rapidly, potentially leading to sepsis, amputation, and death if not recognized early. It has an estimated incidence of 0.24 to 0.4 cases per 100,000 per year in the Western world [1] and a high mortality rate of 25% to 35% [2]. Its presentation is relatively rare in the upper limbs, reporting as low as 6% to 27% compared to the lower limbs without a difference in sidedness or gender predilection [3].

Risk factors include diabetes mellitus, immunocompromised states, alcoholism and morbid obesity. The affected area is usually painful, erythematous, or warm to the touch, symptoms that can lead to misdiagnosis with other dermatological or infective conditions. More specific signs are crepitus, bullae formation and skin necrosis, but these are less common and, therefore, a high level of suspicion must be maintained. Any indication of systemic changes such as fever, hypotension or hyperglycemia would warrant further investigations, mainly if associated with organ failures such as renal failure or hypoxia.

The Laboratory Risk Indicator for Necrotizing fasciitis (LRINEC) is a tool that can be used to differentiate necrotizing from other non-necrotizing soft tissue infections. It focuses on laboratory values such as C-reactive protein (CRP), white cell count (WCC), hemoglobin (Hb), sodium concentration, creatinine and blood glucose levels [4]. A score of 6 or more suggests NF. The SIARI (Site other than lower limb, Immunosuppression, Age <60 years, Renal failure, Inflammatory markers) score is another tool that has been applied in the diagnosis of NF [5]. It utilizes findings on assessment in addition to laboratory parameters of creatinine, CRP levels and WCC.

This case report demonstrates a 60-year-old male presented with pain and swelling to the left forearm and was initially thought to have cellulitis. The paper aims to describe a rare manifestation of NF of the upper limb that masqueraded as a common emergency department (ED) presentation. It highlights the diagnostic challenges involved while comparing the screening tools that can be used to aid in its early recognition.

## Case presentation

A 60-year-old livestock farmer presented to the ED with a six-day history of swelling, pain, and oozing blebs on his left forearm. He had been referred to the hospital by his private physician for upper limb cellulitis after refractory management with antibiotics and steroids. He had no history of trauma or insect bites to the limb and no significant medical conditions. At presentation, he was afebrile and tachycardic (116 bpm) but otherwise stable.

There was a concern about his physical findings being suggestive of a severe case of cellulitis, but the possibility of NF could not be ruled out. As a result, he was referred to the Orthopedic Department and had routine blood investigations done. The results were significant for elevations in the WCC of 27.6 x 10^3/uL and CRP of 253 mg/dL, as well as an altered kidney function.

Table 1 displays the blood investigations obtained for this patient. Other blood markers were within normal range.

**Table 1:** Blood investigation results from the Emergency Department

Blood Investigations	Result/Units	Normal Range
Haemoglobin	11 g/dL	10.80-14.20 g/dL
White cell count	27.6 x 10៱3/uL	3.70-10.10 ៱3/uL
Platelets	195 x 10៱3/uL	155-366 10៱3/uL
C-reactive protein	253 mg/L	0.3-1.0 mg/dL
Creatinine	3.7 mg/dL	0.50-1.20 mg/dL
Sodium	128 mmol/L	136-145 mmol/L
Potassium	4.6 mmol/L	3.50-5.10 mmol/L
Random blood glucose	180 mg/dL	70-110 mg/dL

On review, the Orthopedics Department agreed with a diagnosis of NF, and the patient was promptly prepared for emergency debridement in the operating theatre.

**Figure 1:**
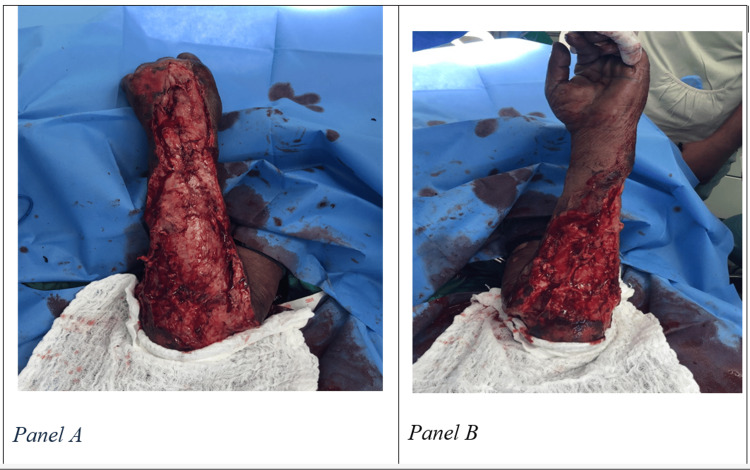
Findings in operating theatre six hours after diagnosis Extensive necrosis of the forearm, primarily on the dorsal aspect (Panel A) with moderate extension into the volar aspect (Panel B).

Six hours later, assessment in the operating theatre showed extensive necrosis of the forearm, primarily on the dorsal aspect with moderate extension into the volar aspect, as seen in Figure 1. All apparent necrotic tissue was debrided, and a tissue sample was sent off for microscopy, culture and sensitivity (MCS) investigation.

Following debridement, he was started on empiric antibiotics, with plans to modify them once sensitivity results were obtained. Wound swabs for MCS were also done intermittently to identify two consecutive 'no bacterial growth' results in preparation for skin grafting, as required by the Plastic Surgery Department.

The MCS from the tissue sample revealed a gram-negative rod identified as *Aeromonas hydrophila*, a bacteria common in low salinity or fresh water and soil. Based on these findings, empiric antibiotics were discontinued, and trimethoprim/sulfamethoxazole was commenced. Table 2 summarizes the organisms found on repeated wound swab MCS and their sensitivities over four weeks.

**Table 2:** Results from microscopy, culture and sensitivity of tissue sample and wound swabs over the admission period

Date	Cultured Organism	Sensitivity
13-01-23	Aeromonas hydrophila	Cefepime, ceftriaxone, trimethoprim/sulphur
26-01-23	Normal skin flora	-
29-01-23	Pseudomonas aeruginosa	Ciprofloxacin, cefepime, gentamicin, amikacin
01-02-23	No bacterial growth	-
03-02-23	No bacterial growth	-
05-02-23	P. aeruginosa	Cefepime, ceftazidime
07-02-23	Normal skin flora	-

The patient required a second debridement a few days after the first procedure. Following each procedure, a honey-based dressing was applied to the affected area of the forearm and changed every two to three days.

**Figure 2:**
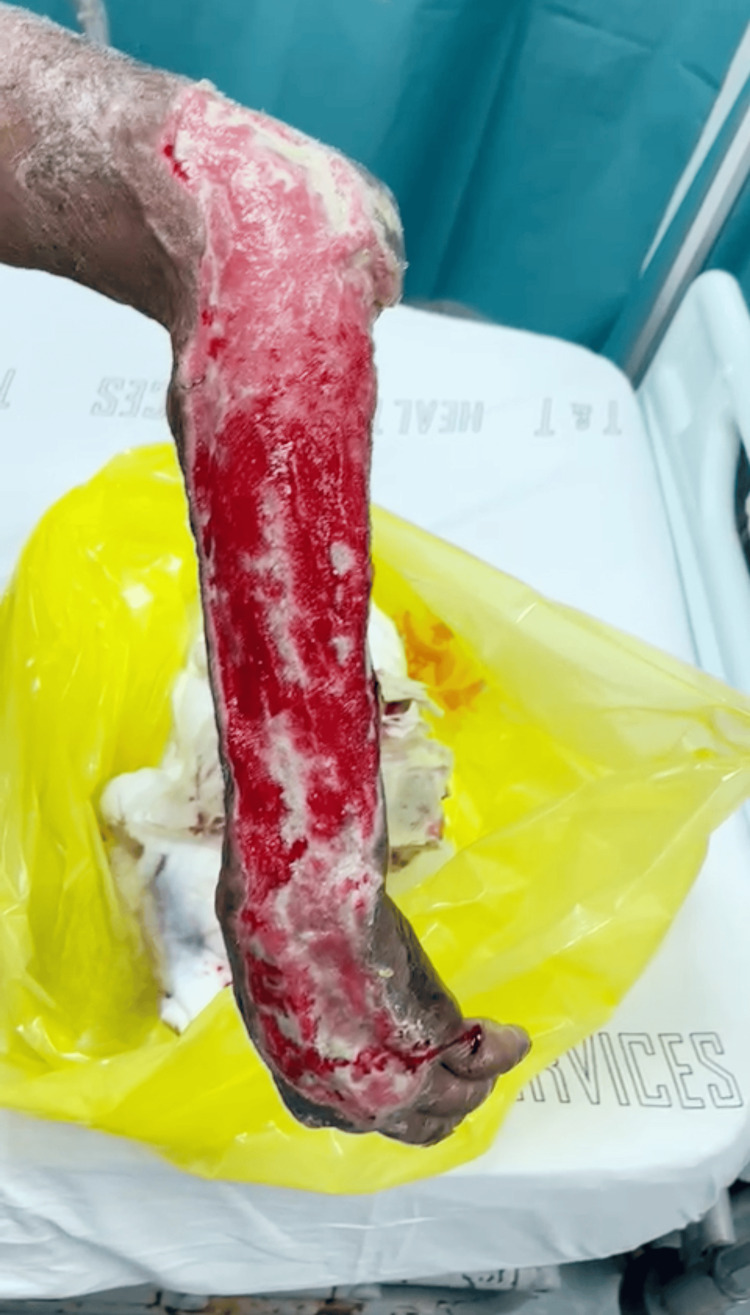
Progress of left upper limb after one week New granulation tissue with generalized slough, particularly at the proximal aspect of the elbow.

One week after the second debridement, new granulation tissue with generalized slough, particularly at the proximal aspect of the elbow, was noted, as seen in Figure 2. The forearm continued to gradually improve over a one-month period.

**Figure 3:**
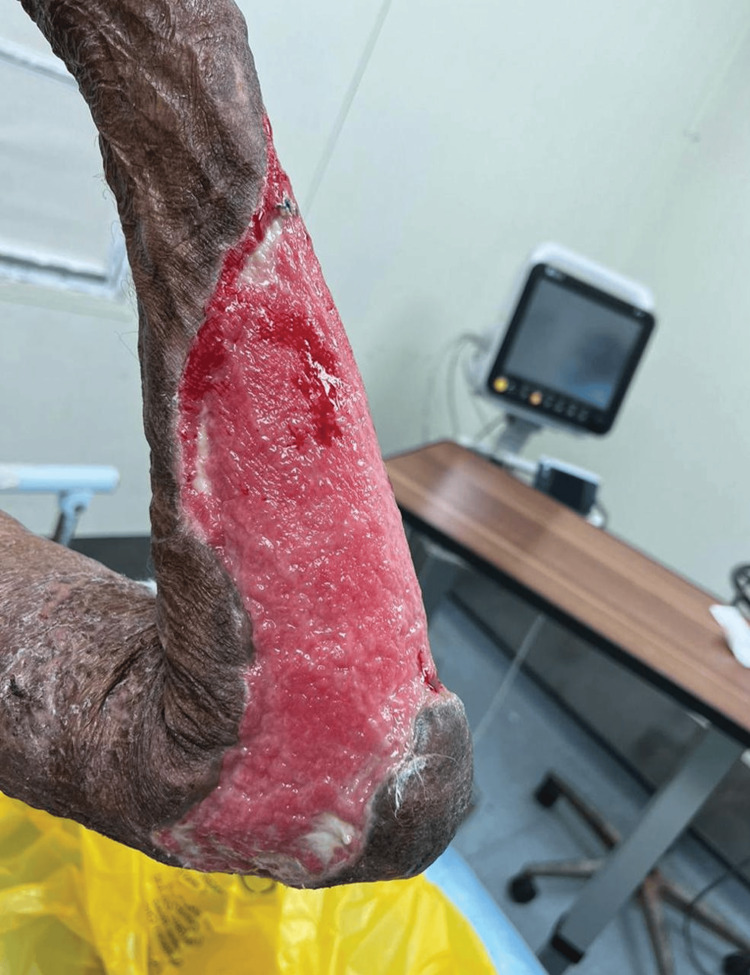
Progress after one month Significant granulation tissue with very minimal slough over the left forearm.

After a month, there was significant granulation tissue with very minimal slough, as seen in Figure 3. After two consecutive 'no bacterial growth' swabs had been achieved, the patient was referred to the Plastic Surgery Department, where he received split skin grafting.

**Figure 4:**
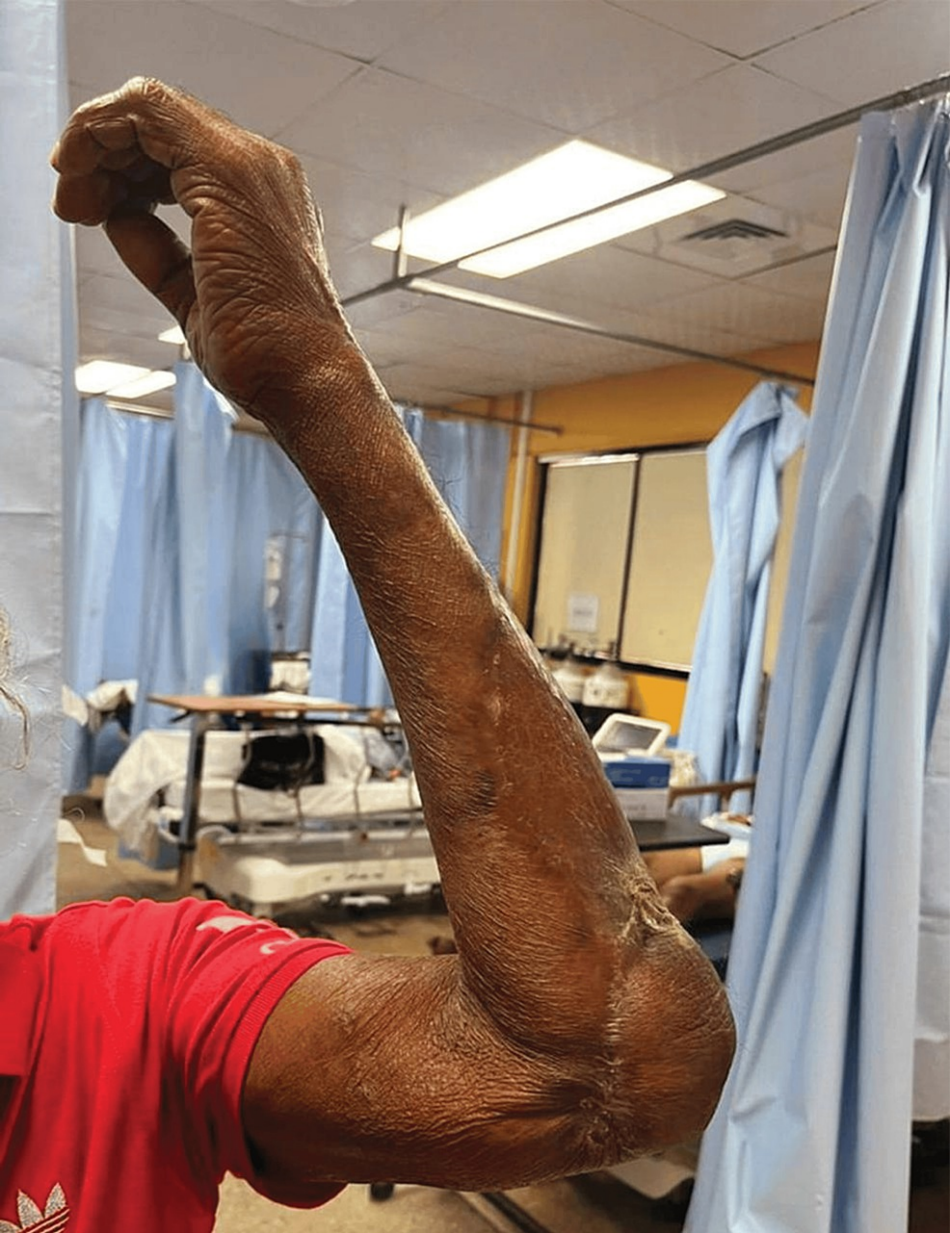
Progress after four months Healthy skin over left forearm with scars indicating split skin grafting.

Four months later, the patient presented for a follow-up at the clinic. Figure 4 shows images of his left upper limb, showing healthy skin over the dorsal and ventral aspects of the left forearm.

The patient spent eight weeks in a medical institution after the diagnosis of left upper limb NF was confirmed. The combination of urgent surgical debridement, IV antibiotics, and skin grafting resulted in him maintaining the limb's functional status. He is still employed as a farmer and has no limitations to his daily activities.

His diagnosis at presentation to the ED catalyzed an urgent multidisciplinary team approach involving co-management with Orthopedics, Microbiology and Plastic Surgery, which fortunately resulted in a favorable outcome.

## Discussion

The Centre for Disease Control (CDC) defines NF as a bacterial infection (more commonly known as 'flesh-eating disease') that has the potential to progress to septic shock and multi-organ failure if untreated [6]. It can be classed into four types. Type 1 is poly-microbial, type 2 is mono-microbial (usually due to group A streptococcus), type 3 is linked to marine exposure and type 4 involves organisms seen in immunocompromised individuals [7].

The pathophysiology behind this disease involves the offending bacteria entering the body through a break or disruption in the skin barrier. Once under the skin, these organisms can release exotoxins that will instigate a significant inflammatory response by activating immune mediators [7]. The resultant thrombosis of the dermal papilla results in a darkish discoloration of the skin [8].

Some common signs and symptoms of NF are the affected area being erythematous, swollen, warm, and highly tender, especially in the initial stages [1,2]. Later, hemorrhagic blisters are formed due to skin necrosis [8]. These were all signs and symptoms that the patient in this case had; however, his physical findings were initially believed to be more in keeping with cellulitis.

A delay in diagnosis can occur due to manifestations of NF being comparable to other cutaneous conditions. Besides cellulitis, it can also be mistaken for insect bites, pressure ulcers or gas gangrene [9]. Interestingly, an accurate admitting diagnosis of NF only occurs approximately one-third of the time in cases [8]. In a retrospective study in the United Arab Emirates (UAE) in 2007 by Hefny et al., it was found that out of 11 patients presenting to the hospital over two years with NF, seven were initially misdiagnosed [10]. Other studies have also noted that in approximately 85% of cases, NF is not considered as the initial diagnosis (Wei et al., 2023) [11]. This ambiguity can manifest if clinical judgment alone is used to make a diagnosis. For that reason, recognizing this acute presentation to an ED as a surgical emergency is critical, and this is where scoring tools can prove beneficial.

Over the years, clinical predictor tools have been increasingly used in EDs. Many of them have proven helpful in expediting patient care and contributing to shortening treatment delays. There are two predictor scores for NF: the LRINEC and SIARI scores.

The LRINEC scoring criteria were created in 2004 by Wong et al. during a retrospective observational study in Singapore [12]. The score involves using six blood tests and investigations whose parameters can guide a patient's clinical status with suspected NF. The study consisted of 140 patients with NF who had investigations done on presentation and logistic regression used to identify significant clinical predictors [12,13]. It found that a score of six and over had a positive predictive value (PPV) of 92% and a negative predictive value (NPV) of 96% for NF [14].

The investigations identified were the WCC, Hb, sodium, creatinine, CRP, and glucose. These lab studies can all be quickly obtained within an ED with functional and active laboratory services or stat blood test machines.

The SIARI score stands for: Site other than the lower limb, Immunosuppression, Age < 60 years, Renal impairment (creatinine > 141), and Inflammatory markers (CRP ≥ 150, WCC > 25] [15].

This score was developed in New Zealand, and findings were published by Cribb et al. in 2019. The six predictor categories were determined from analysis of patient demographics and physical and laboratory findings within their patient population. This tool utilizes a quicker assessment and requires fewer parameters, making it easy to obtain a prediction score. In its validation study of 138 with NF, the SIARI score was found to have a sensitivity of 84% (95% CI 74-92%) and specificity of 70% (95% CI 57-80%) [15].

Tables 3-4 compare the application of both scoring systems with the results and findings of the patient from this case presentation.

**Table 3:** Laboratory Risk Indicator for Necrotizing fasciitis parameters with patient’s results

Parameter	Range	Score	Patient's Value
White cell count (per mm^3^)	<15	0	-
	15-25	1	-
	>25	2	27.6
Hemoglobin (g/dL)	>13.5	0	-
	11-13.5	1	11.41
	<11	2	-
Sodium (mEq/L)	≥135	0	-
	<135	2	126
Creatinine (mg/dL)	≤1.6	0	-
	>1.6	2	3.7
C-reactive protein mg/L	<150	0	-
Glucose (mg/dL)	>150	4	253
	≤180	0	-
	>180	1	180
Composite Score	Score	Interpretation	-
	<6	Low risk	-
	06-Jul	Intermediate risk	-
	≥8	High risk	Patient score: 12

**Table 4:** SIARI (Site other than the lower limb, Immunosuppression, Age <60 years, Renal failure, Inflammatory markers) score with patient’s results

Variable	Score Attributed	Patient's Values
Site of infection outside of the lower limb	3	3
History of immunosuppression	3	0
Age ≤60 years	2	1
Creatinine >141 umol/L	1	1
White cell count >25 per mm^3^	1	1
C-reactive protein ≥150 mg/L	1	1
	Maximum score: 11	Patient score: 7

Utilizing either scoring system would have categorized this patient as high risk for NF, with the LRINEC score indicating a higher likelihood based on its parameters.

The application of the LRINEC score has been helpful in many clinical settings. A 2022 retrospective study by Hoesl et al. of 70 patients in Germany with NF found that when the LRINEC score was applied, it proved to be an important prognostic marker for lethality in patients with a score of more than 7 [4]. Additionally, a systematic review in the United Kingdom of 18 case publications from 2004 to 2018 on the reliability of LRINEC score for NF by Abdullah et al. (2019) found it to be a dependable tool in identifying high-risk patients [16]. A meta-analysis in the United States of the score in predicting upper and lower extremity NF by Tarricone et al. (2022) also found it helpful in differentiating NF from other soft tissue infections [17].

However, in comparing the two studies, Crib et al. (2019) found the SIARI score to show greater diagnostic accuracy than the LRINEC score in cases of NF [15]. Supporting this was a prospective study by Kishor et al. (2022) of 32 confirmed cases of NF. They found that the SIARI score also showed better diagnostic accuracy as well as higher specificity (70%) and sensitivity (78.1%) than the LRINEC score (sensitivity of 62% and specificity of 60%) [18].

It must be mentioned that these criteria have limitations in their applications. A 2017 systematic review by Bechar et al. in England found two reports of patients with NF but LRINEC scores of less than 6, indicating they would be classed as low risk [19]. Similarly, in a retrospective analysis in Australia by Holland et al. studying 28 patients who were admitted for NF and had the LRINEC score applied, it was found that it had a sensitivity of only 80% and a specificity of 67%, allowing for some cases to be missed [20]. There are, however, few publications comparing the accuracy of the SIARI score application.

Regardless, early application of either score could have proven more beneficial for the patient than clinical judgment alone. Differentiating physical findings can be challenging in the ED and may vary with years of experience. Hence, calculating the patient's score using either system would have identified him as a high-risk patient for NF, and immediate consultation could have been obtained. This calculation is essential when considering that there is a window for debridement within 12 hours that, if missed, can contribute to the increased morbidity and mortality associated with NF [2].

Ultimately, this patient received what is considered the mainstay treatment for NF. He had surgical debridement of the necrotic tissue of his upper limb coupled with IV antibiotics. The patient went on to have a favorable outcome. However, this might not have been if there had been any further delays. No further validation studies exist for LRINEC or SIARI scoring systems, but more will likely develop with time. In the interim, using these predictor tools, along with clinical experience and acumen, can ensure that cases of NF are identified promptly and delays in management are avoided.

## Conclusions

The presentation of NF can overlap with many other medical ailments of varying severity, making it easy to miss. Therefore, it is crucial to use high clinical suspicion and scoring tools such as LRINEC and SIARI to rule out the likelihood of NF.

The superiority of the scoring systems when compared indicates that the SIARI tool may have a higher sensitivity when compared with that of the LRINEC. Though said clinical tools have yet to undergo many validation studies, this case report and prior studies show they can aid with rapid diagnosis, leading to prompt management. With further investigations and the development of additional clinical predictor tools, more life-threatening diseases like NF will likely be quickly recognized from the point of entry to the ED, and the involvement of specialty services and effective care will be expedited.
